# Investigation of changes in bone density and chemical composition associated with bone marrow oedema-type appearances in magnetic resonance images of the equine forelimb

**DOI:** 10.1186/s12891-019-2693-y

**Published:** 2019-07-15

**Authors:** Christine J. Heales, Ian R. Summers, Jonathan Fulford, Karen M. Knapp, C. Peter Winlove

**Affiliations:** 1 College of Medicine and Health, University of Exeter, St Luke’s Campus, Heavitree Road, Exeter, EX1 2LU UK; 2 College of Engineering, Mathematics and Physical Sciences, University of Exeter, Streatham Campus, Stocker Road, Exeter, EX4 4QL UK

**Keywords:** Magnetic resonance imaging, Osteoarthritis, Bone remodelling, Raman, Bone density, Bone marrow oedema

## Abstract

**Background:**

The aetiology of bone marrow oedema-like abnormalities (BMOA) seen on magnetic resonance imaging (MRI) is as yet not fully understood. The current study aimed to investigate the potential of projection radiography and Raman microspectroscopy to provide information regarding the underlying physiological changes associated with BMOA in equine bone samples.

**Methods:**

MRI was used to assess 65 limbs from 43 horses. A subset of 13 limbs provided 25 samples, 8 with BMOA present and 17 as controls; these were examined with projection radiography to assess bone mineral density and Raman spectroscopy to assess bone composition. Statistical analysis was conducted using SPSS, the relationship between BMOA and age was tested using binary logistic regression, other outcome measures via unpaired *t-*tests.

**Results:**

Overall BMOA was found to be associated with locally increased bone density (*p* = 0.011), suggesting increased bone formation; however, no measurable changes relating to bone remodelling were found, and there were no detectable changes in the chemical composition of bone.

**Conclusions:**

BMOA is associated with locally increased bone density, without an associated change in the chemical composition of bone, suggesting this is not linked to BMOA. The presence of increased bone density associated with BMOA does appear to suggest that an increased amount of bone formation is occurring in these regions, but as Raman microspectroscopy data do not demonstrate any significant changes in bone chemical composition associated with BMOA, it would appear that the increased bone volume is due to a greater amount of bone being formed rather than an imbalance in relation to bone remodelling.

The study provides a proof of principle for the use of Raman microspectroscopy and projection radiography in *in vitro* studies of BMOA.

**Electronic supplementary material:**

The online version of this article (10.1186/s12891-019-2693-y) contains supplementary material, which is available to authorized users.

## Background

Bone marrow oedema-like lesions seen on magnetic resonance imaging (MRI) tend to be clinically non-specific in appearance, and thus it is suggested such lesions be referred to as a bone marrow oedema-like abnormality (BMOA) [1]. Excluding those related to trauma [2], BMOAs in humans are associated with a range of conditions including diabetic neuropathy [3], osteoarthritis, rheumatoid arthritis [1, 4] and osteomyelitis [3].

As yet, the aetiology of non-acute trauma BMOA within bone is not fully understood. BMOA has been associated with pain, even in the absence of direct, acute trauma [1, 5], although BMOA has also been found in both asymptomatic individuals [6] and asymptomatic athletes [7]. It has been demonstrated that oedema-like appearances occur when sedentary individuals start a running regime, [5, 6] and these appearances are generally more common in runners than non-runners [7]. In these cases, it has been suggested that the BMOA patterns might represent the early stages of a stress fracture [8], in which case the distinction between BMOA (non-traumatic) and bone-bruising (traumatic) may be less distinct. BMOA has also been described in association with a transient form of osteoporosis, generally affecting a single bone and concluding with restoration of the bone mineral density [9].

Although the clinical significance of BMOA remains unclear, it is nevertheless becoming increasingly pertinent in the investigation of osteoarthritis. It has been shown that, for individuals with osteoarthritis of the knee, the majority of subchondral cysts develop from within regions of bone marrow with oedema-like MRI signal [4, 10] whose presence also relate to the severity of symptoms, degree of cartilage degeneration and disease progression [11–13]. Similarly, the Multicentre Osteoarthritis Study (MOST) demonstrated that subchondral BMOA lesions are highly associated with, and predictive of, bone attrition in individuals who subsequently develop osteoarthritis [14].

Thus, although BMOA is increasingly being regarded as an important aid for the differential diagnosis and subsequent disease management of osteoarthritis [10], [14–16], further research is required to investigate the underlying relationship between BMOA and the physiological changes which underpin the MR image appearance. Previous studies have shown a range of features associated with the presence of BMOAs including bone marrow necrosis, bone marrow fibrosis, reduced mineral density [1, 4] and altered trabecular morphometry [17]. Following on from these studies, the aim of the current study was to investigate BMOA in a range of equine samples measuring bone density via projection radiography and the chemical composition of bone by Raman microspectroscopy.

## Methods

### Study samples

**Fig. 1:**
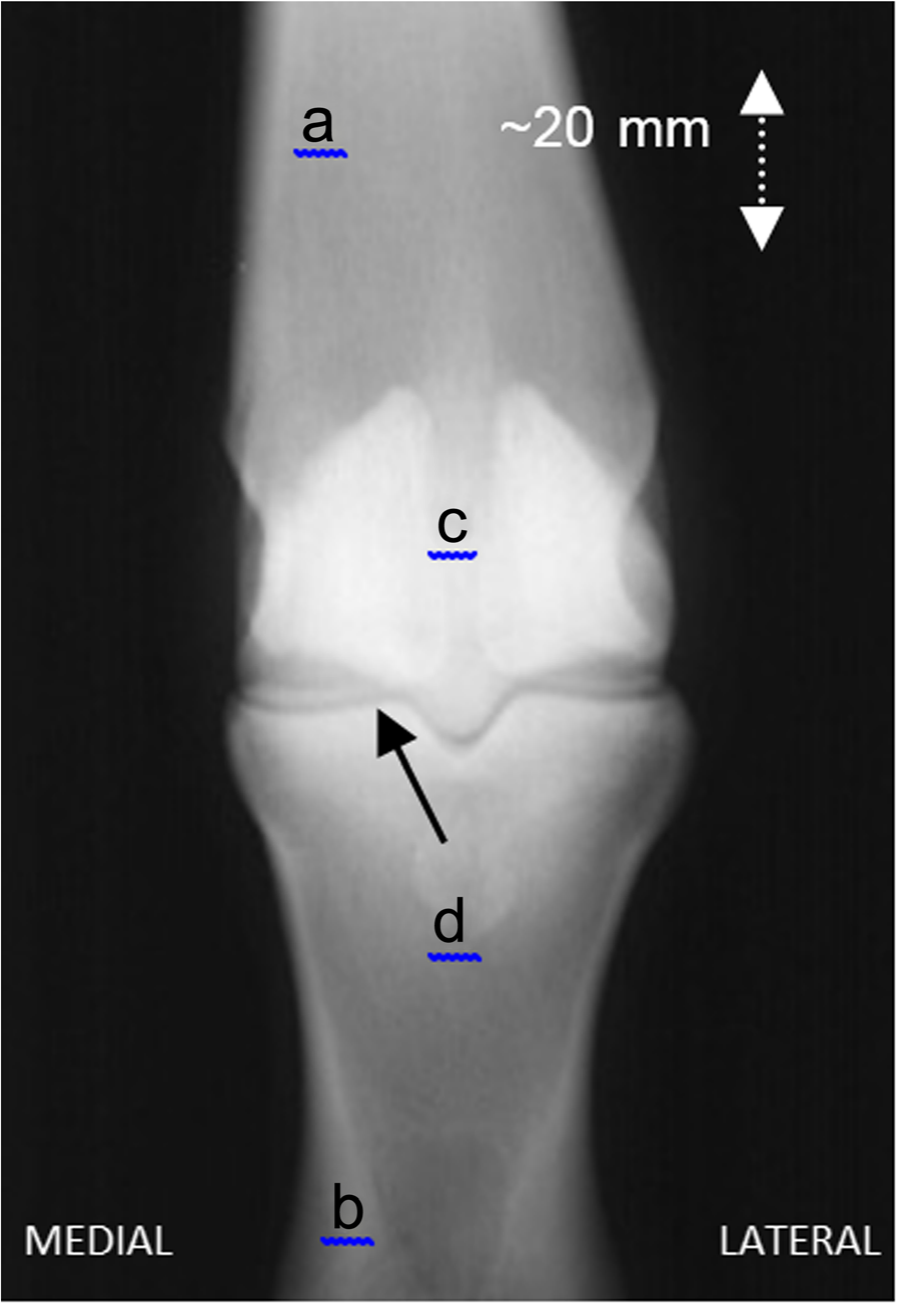
Dorso-palmar (antero-posterior) radiograph of the metacarpophalangeal joint. Key: a third metacarpal bone, b proximal phalanx, c proximal sesamoid bones, d metacarpophalangeal joint

Bone samples were obtained from a local abattoir, where all horses were being euthanized for humane reasons. Sixty-five samples (some from a single forelimb, some from both forelimbs, dependent on availability), were collected from 43 horses, the working histories of which were not known, although observation of the animals prior to euthanasia indicated a mixed pedigree; yearlings, riding school horses and ponies, as well as wild Dartmoor ponies. Wherever possible (*n* = 34), the animals were aged by experienced abattoir staff (age: mean ± s.d.: 13.4 ± 5.4 years); this provides an estimation of age, although it is not an entirely precise method [18]. The samples obtained were of the distal third metacarpal bone of the equine forelimb (Fig. 1), which is a high-load, high-velocity joint, comprising an articulation between the distal end of the third metacarpal bone, the proximal phalanx, and a further articulation between the palmar surface of the metacarpal and the two proximal sesamoid bones.

### MRI imaging

**Fig. 2:**
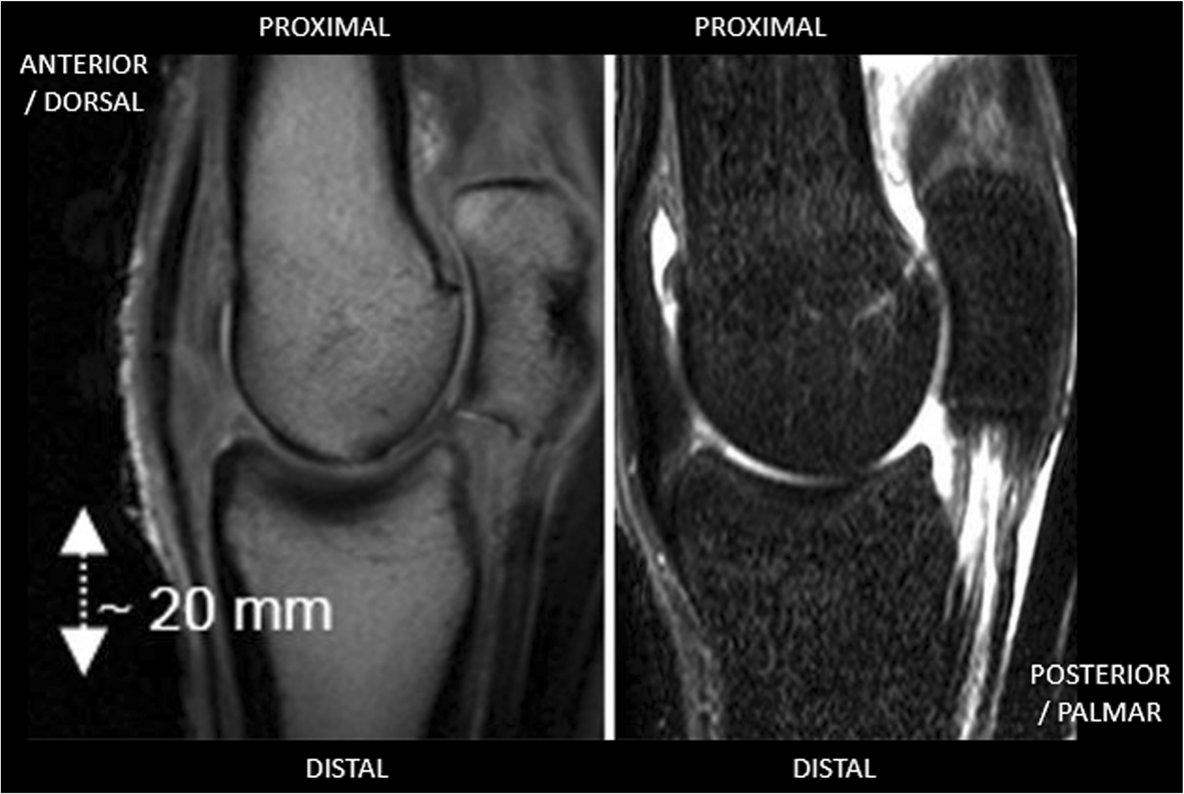
Parasagittal magnetic resonance images of the metacarpophalangeal joint. T1w image on left, STIR image on right, no evidence of BMOA

**Fig. 3:**
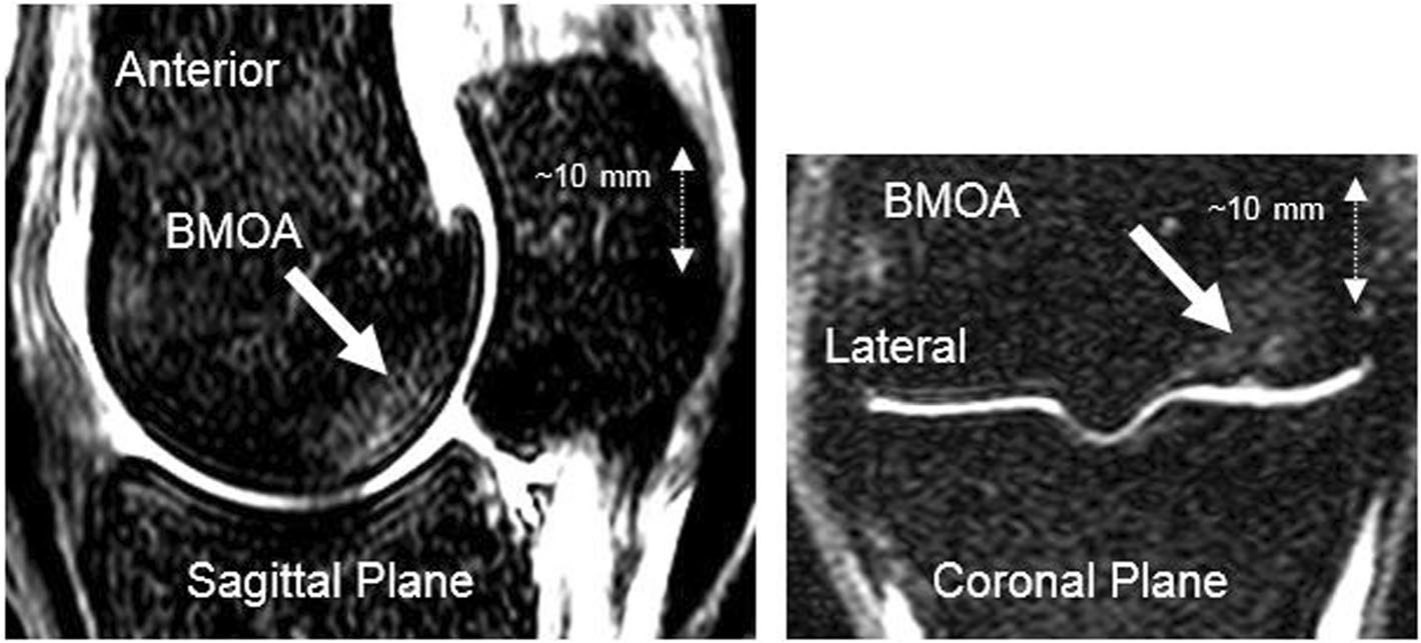
Coronal and midline sagittal magnetic resonance images of the metacapophalangeal joint. Arrows indicate the region of the BMOA

MRI imaging was undertaken on fresh, refrigerated, limbs between 4 and 6 h after the samples were obtained. The imaging was undertaken using a Philips Intera 1.5 T scanner (Philips, NV) and a two-element small flex coil. Scans obtained consisted of short tau inversion recovery (STIR) sequences (repetition time TR 4475 ms, echo time TE 9 ms, inversion delay TI 150 ms, slice thickness 3 mm, acquired resolution 0.59 × 0.85 mm), T1 weighted sequences (TR 35 ms, TE 5 ms, slice thickness 2 mm, acquired resolution 0.59 × 0.76 mm) (Fig. 2) and T2 weighted sequences (TR 4220 ms, TE 95 ms, slice thickness 3 mm, acquired resolution 0.49 × 0 .62 mm). All scans were obtained in the sagittal plane, aligned with and parallel to the median sagittal ridge of the distal third metacarpal bone. In addition, coronal STIR images were occasionally taken to aid the localisation of specific bone marrow oedema lesions (Fig. 3).

**Fig. 4:**
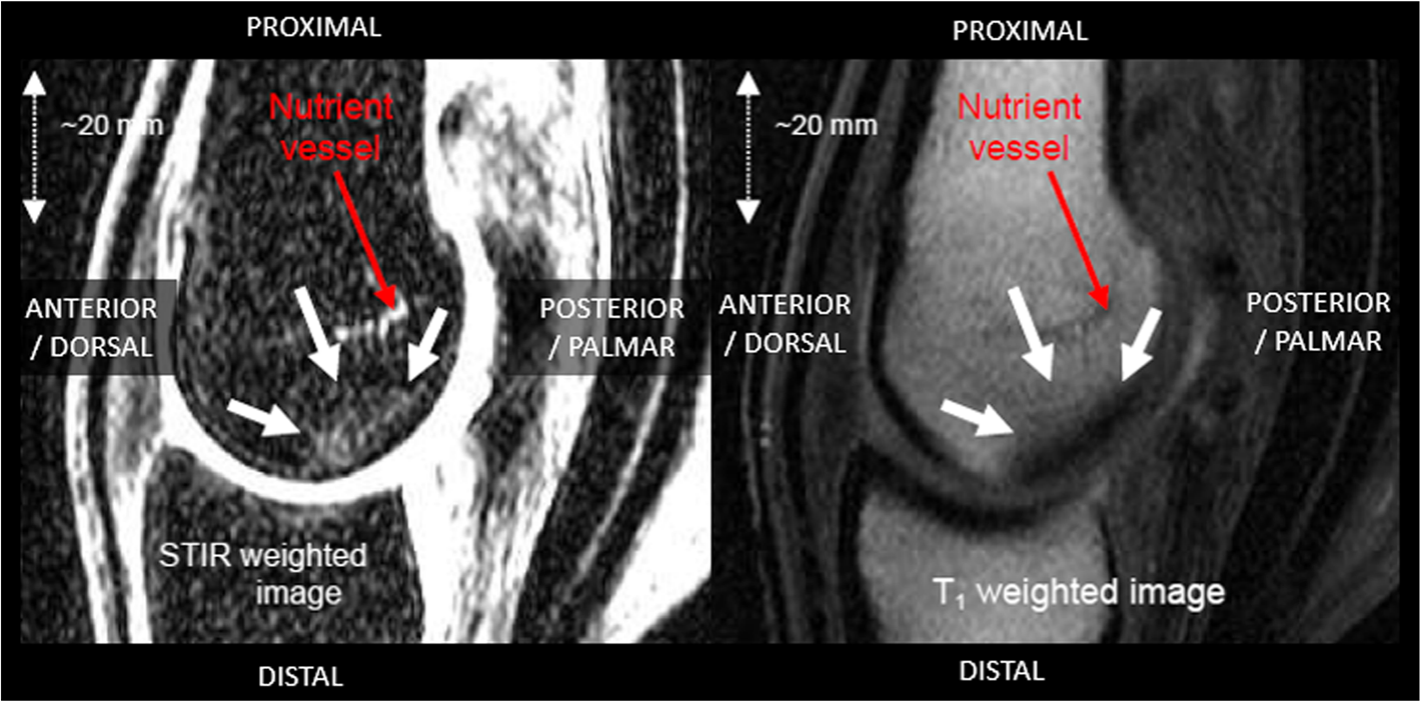
Mid-sagittal magnetic resonance images of the metacarpophalangeal joint. Arrows indicate the region of the BMOA

Image evaluation was undertaken by a single, experienced MRI radiographer (author CJH) who also performed the image acquisition. BMOA was defined as a region of high signal (hyperintensity) on STIR images and corresponding low signal (hypointensity) on T1 weighted images [17] (Fig. 4) due to oedema or interstitial fluid within the extracellular spaces of the bone marrow [19]. T1 and T2 sequences also allowed the identification of acute trauma from features such as fracture lines, soft-tissue oedema, swelling or haemorrhage as well as demonstrable pathology such as limb deformity or advanced osteoarthritis that would mean samples would be excluded from the study. As a result, three samples were excluded as the BMOA detected was felt to be due to acute trauma (the location of the altered signal intensity being suggestive of extreme extension of the joint), a blood vessel and a cyst, respectively. The sample with the cyst was excluded as it may have been representative of advanced osteoarthritis or been a unicameral or aneurysmal cyst and may have confounded the analysis. The 65 limbs were then divided chronologically into subsets for pilot and other studies. Thirteen limbs were selected for subsequent analysis within the present study. Each limb provided two sample slices (medial and lateral – see below), 8 with BMOA present (mean age ± sd 16.0 ± 4.1 years) and 17 controls (mean age ± sd 17.2 ± 4.1 years). One sample slice was excluded due to the presence of a cyst (see above). Of the 8 slices with BMOA present, six were obtained from limbs with both medial and lateral BMOA, and two from limbs with medial BMOA only. These 25 sample slices were subsequently investigated by Raman spectroscopy and projection radiography.

### Raman microspectroscopy

**Fig. 5:**
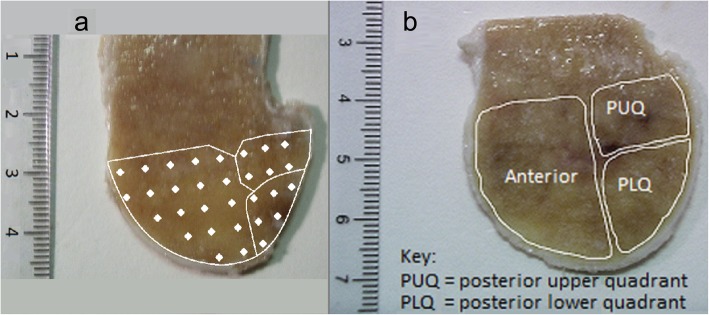
Photographs of parasagittal (medial) bone section. **a** represents sampling pattern for locations of individual Raman microspectroscopy measurements (indicative only) and, **b** regions of interest used for Raman microspectroscopy and bone density measurements

Raman microspectroscopy was performed on a subset of animals, after pilot studies to ensure adequate sample preparation. Following MRI scanning and the identification of areas of BMOA, the distal portion of the third metacarpal bone was dissected. Soft tissues and ligaments were removed (Fig. 5) and two 1 mm slices through the bone were cut along the sagittal plane, on either side of the midline, passing through the mid-region of the BMOA lesion (when present) or in a corresponding location (typically 10 mm from the midline) when BMOA was not present. The majority of BMOA lesions, and all eight BMOA lesions in the selected subset (see below), were found in the palmar condyles, the region of greatest loading in the metacarpal [20] and hence all control bone sections were taken from the same site.

Bone slices were fixed in 10% weight by volume (w/v) formalin for 24 h, rinsed thoroughly in 0.9% w/v saline, and stored under 0.9% w/v saline. Raman microspectroscopy was undertaken within a week of sample preparation, typically within 24 h. Prior to undertaking Raman microspectroscopy, the surface of each slice of fixed bone was polished with glass paper (firstly 800 grade, then 1200 grade) to ensure a uniform, smooth surface, and then cleaned ultrasonically in a 0.9% w/v saline bath for 30–60 s using a Sonic3000SS Professional (UK) ultrasonic cleaner. This procedure ensured a smooth, debris-free surface, thereby maximising the amount of scattered light received by the Raman microscope and thus optimising the quality of the Raman spectrum generated [21].

Raman microspectroscopy was undertaken using a Renishaw 1000 Raman Microscope system (Renishaw, UK), utilising excitation from a 100 mW helium-neon laser, at a wavelength of 785 nm. Prior to each data collection session the Raman microscope was calibrated using a wafer of silicon (expected wave number of 520 cm^− 1^). For the measurements of bone slices, a × 40 microscope objective was used, yielding a field size in-plane of around 10 μm × 10 μm. Measurements of each sample were obtained at multiple individual points to provide average data which were unaffected by any local variations within the bone. Between measurements the sample was moved approximately 3 mm using a micrometer stage in either the *x* or *y* direction such that sampling had an approximate grid pattern (see Fig. 5a for schematic representation of sampling strategy). The spacing between measurements was not exactly 3 mm because the sample position was adjusted on the sub-millimetre scale to ensure the laser was focused on the extracellular matrix (that is mineralised bone). At each location there were two 10-s data acquisitions. The spectra obtained covered a range of Raman shifts (Δώ) from 500 cm^− 1^ to 3000 cm^− 1^.

**Fig. 6:**
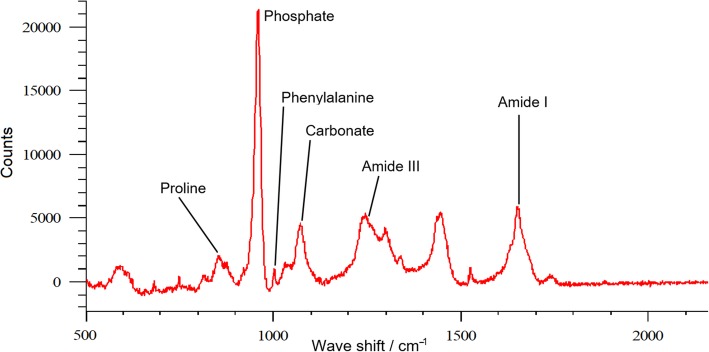
Expanded portion of Raman spectrum demonstrating position of peaks of interest

Following acquisition, the spectra acquired at each individual location were pre-processed and analysed; a representative spectrum is provided in Fig. 6. Initially, baseline correction was undertaken using the software package provided as part of the Renishaw system (WiRE 2.0). The areas under the peaks described in Table 1 were subsequently determined using the curve fitting function provided within the Renishaw software.

**Table 1:** Peaks quantified within Raman spectra

Chemical Bond/Molecule	Approximate Peak Centre (cm^-1^)	Range to which peak area was fitted (cm^-1^)
Proline	855	830 to 870
Phosphate (ν1-phosphate band)	956	925 to 980
Phenylalanine	1003	990 to 1010
Carbonate (ν1-carbonate mode)	1071	1050 to 1095
Amide III	1240, 1270	1200 to 1300
Amide I	1665	1565 to 1720

Previous studies in bone have demonstrated that Raman microspectroscopy can determine the changes in bone chemical composition that occur with fracture healing [22], with aging [23] and with osteoporosis [24]. However, it has also been shown that fixation may lead to alterations in specific peaks within the Raman spectra [25]. In the present study following peak quantification a range of measures was determined, as follows:

1. Peak centre value of phosphate to examine crystallinity [26].
2. For an indication of the mineral:matrix ratio, the ratio of peak areas for phosphate:amide I and carbonate:amide I [26, 27].
3. The ratio of carbonate:phosphate to examine type B carbonate substitution [27].

Following analysis of the individual-location spectra, data were averaged over specific regions of interest within the sample slices. Location names are given such that anterior would correlate to the dorsal location in the horse, posterior to palmar, upper to proximal and lower to distal anatomical locations. The sample locations were identified as the anterior, the posterior upper quadrant (PUQ) and posterior lower quadrant (PLQ), as shown in Fig. 5. The lesion, when present, was within the PLQ, and the area of the PLQ was defined such that it encompassed all of the lesion with a border in the order of 2–3 mm. A within slice ratio (WSR) was subsequently calculated for each of the parameters, i.e., the ratio of the average value in the PLQ region to the average found in the anterior and PUQ regions. This was in order to reduce the influence of the heterogeneity of the sample group: variations of bone composition between animals due to factors such as age, working history and breed, effectively normalising the PLQ region to the non-lesion area of bone for each animal.

### Bone density measurements

Following Raman microspectroscopy, projection radiographs were taken of the same subset of bone slices using a Siemens Multix Top (Siemens, Munich) ceiling mounted x-ray system in conjunction with a Konica Regius 150 (Konica Minolta, Tokyo) computed radiography system, using the following exposure parameters: source to image receptor distance (SID) 115 cm, fine focus (0.6 mm focal spot), 50 kVp and 1 mAs. The images were processed using a Konica Regius 150 pre-programmed fixed linear look-up table.

The assessment of bone mineral density (BMD) was undertaken within the same regions of interest as identified for the Raman microspectroscopy, namely the anterior, PUQ and PLQ. A miniature aluminium step-wedge (7 steps, each 0.5 mm in depth) was included in all projection radiographs undertaken to enable the calculation of the BMD of each area/sample in terms of mm of aluminium equivalence. Aluminium has an atomic number of 13, and the effective atomic number of bone has been cited of the order of 11.6–13.8 [28]. Hence, for the purposes of normalisation, it was assumed that the relationship between thickness of aluminium and image intensity was comparable to the relationship between thickness of bone and image intensity. This yielded a dataset consisting of image intensity values which were then converted into mm of aluminium equivalence in order to enable meaningful comparison of bone volume to be made. In order to correct for any variation in the thickness of different bone samples and across the sample, multiple thickness measurements were taken for each sample using a micrometer, and the results averaged. The average number of measurements within each region were: PLQ 6 (range 5–9), PUQ 3 (range 2–5), Anterior 6 (range 5–9). Each BMD was then corrected such that it represented the BMD per mm of bone as an aluminium equivalence.

A WSR was subsequently calculated equal to the ratio of the BMD found in the PLQ region of interest to the average of the BMDs found in the anterior and PUQ regions.

### Data analysis

Statistical analysis was conducted using SPSS version 22.0 (SPSS Armonk, NY). Results quoted are given as mean ± standard error. To assess the relationship between BMOA presence and age, binary logistic regression was run. Given the lack of demographic information, no other variables were included wtihin the analysis. For Raman outcome measures testing was undertaken to examine whether WSRs were different for sample slices with and without BMOA via unpaired *t*-tests. To assess whether there was a general tendency for PLQ bone density to be higher than the rest of the sample, BMD data from all samples were combined, and a 1-sample t-test run to examine whether WSR values were greater than unity. Subsequently WSR BMD values were compared for the BMOA and control groups via an unpaired t-test.

## Results

### Prevalence of BMOA

BMOA was present in 19 horses out of a total of 43 (44%) from which forelimbs were utilised. Of those 22 horses which had both forelimbs scanned, only two (9%) had evidence of BMOA bilaterally in the forelimbs. Of a total of 65 forelimbs scanned, 21 (32%) had evidence of BMOA. From binary logistic regression no significant relationship between BMOA presence and age was found (B = − 0.033, ExpB = 0.967, Wald = 0.228, *p* = 0.630).

### Anatomical location of BMOA

The anatomical location of the BMOA was, in the majority of cases (18 out of 21 forelimbs), on the palmar (posterior) surface, approximately 5–10 mm proximal to the transverse ridge and approximately 5–15 mm from the sagittal ridge with lesions being demonstrated both medially and laterally. Three BMOA lesions were found in atypical locations compared to the majority of lesions. The atypical locations were as follows; the dorsal surface of the epiphysis, central within the distal diaphysis of the metacarpal and on the palmar surface but more superior / proximal. These samples were subsequently excluded. A number of limbs (4 or approximately 19%) had BMOA lesions on both sides of the sagittal ridge. Only one BMOA lesion had an associated cartilage lesion that was demonstrable on the MRI scans. This lesion penetrated the subchondral bone and had an associated subchondral bone deformity.

### Raman microspectroscopy

For the subset of samples for which Raman microspectroscopy and projection radiography were undertaken (BMOA group *n* = 8, control group *n* = 17), an average of 66 (range 39–166) separate Raman microspectroscopy measurements were taken per sample, of which an average of 29.5% were in the PLQ. For the WSR values for phosphate peak centre, the difference between BMOA and control groups (1.0001 ± 8.23 × 10^− 5^, 0.9999 ± 8.77 × 10^− 5^, respectively, *p* = 0.213) was not significant at the *p* = 0.05 level, i.e., even without a statistical correction for multiple comparisons.

Similarly, no significant differences were found between the BMOA and control groups for phosphate:amide I (1.071 ± 0.037, 1.042 ± 0.084 respectively, *p* = 0.752), carbonate:amide I (0.950 ± 0.060, 0.914 ± 0.052 respectively, *p* = 0.658) or carbonate:phosphate (0.845 ± 0.086, 0.866 ± 0.075, *p* = 0.857) (Additional file 1 provides data for individual samples).

### Projection radiography

When control and BMOA groups were combined, WSR values significantly greater than 1 were found (1.132 ± 0.039, *p* = 0.003) suggesting a regional bone density variation, with greatest bone density in the PLQ region of interest, the region associated with the greatest loading on the joint. When comparing BMOA and control group WSR, there was a statistically significant difference between the ratios (1.244 ± 0.029, 1.079 ± 0.052 respectively, *p* = 0.011) indicating a higher bone density in the PLQ region associated with the presence of BMOA (Additional file 1 provides data for individual samples).

## Discussion

Of the 65 samples examined approximately a third were found to have BMOA lesions present. When regions of interest were defined within the samples, no significant differences in the variation between the regions were found when using Raman microspectroscopy to compare BMOA and control samples. However, equivalent comparisons revealed a significant difference when bone mineral density was examined, suggesting an association between BMOA and underlying bone density changes.

### Location of the BMOA lesions

It is of interest that the majority of the BMOA lesions observed were in a very specific location, corresponding to the region of greatest loading within the joint and which is associated with injury and lameness in racehorses [29, 30]. High levels of training amongst young horses, such as race-horses, have been shown to be associated with micro-fractures at high-strain sites including the dorsal third metacarpal [30] and it does appear likely that the apparently characteristic location of the BMOA is related to the loading upon the joint. This may be linked to traumatic damage in some way, even in the absence of clear damage to the articular cartilage, although there was no evidence of microfracture within the samples studied.

### Bone density

The data yielded by projection radiography show that bone density relative to the rest of the slice is increased at sites with BMOA in a way that is not observed at equivalent sites in samples where BMOA is absent. This mirrors findings within a clinical study of 268 human subjects where BMOA lesions in the knee at the site of greatest loading were correlated with increased local bone mineral density [31] and more recent work in the human tibia that has demonstrated an association between BMOA and thickened trabeculae that are increased in number and with less spacing [32]. Given that changes in the hydrostatic pressure of bone marrow may affect the stem and progenitor cells present within bone marrow altering the homeostasis of bone [33] it is possible that BMOA is associated with altered hydrostatic pressure in bone marrow and that the changes in bone density observed are a reflection of increased bone deposition. However, further work is required to examine this hypothesis.

### Bone composition

The Raman microspectroscopy measurements related to a range of bone composition characteristics, indicating crystallinity, mineral:matrix ratio, and type-B carbonate substitution. Results showed no significant differences in WSR between samples where BMOA was present and samples where BMOA was absent. The conclusion is thus that BMOA is not associated with modification in bone composition.

It has been demonstrated both in vitro [34] and in vivo [35, 36] that the phosphate band centre shows a positive shift with bone maturity due to increasing tissue age rather than animal age [37]. That the WSRs for this parameter showed no significant difference between BMOA samples and controls is thus indicative of there being no difference in the proportion of immature bone present and thus no difference in the amount of remodelling taking place at BMOA sites.

Changes in mineral:matrix ratio with age have previously been observed in humans with no known bone disorders [33], and may be related to changes in the remodelling rate. Changes in matrix component with age (indicated by the amide I component) have also been observed [23]. A study of collagen structure of normal human trabecular iliac bone using chemical analysis [38] demonstrated a reduction in the amount of collagen with age. In the present study, no mineral: matrix ratio changes were detected in the BMOA region compared to the rest of the sample slice, suggesting that BMOA is not associated with either altered mineral or matrix composition.

Previous Raman studies of bone have principally focused on cortical bone, both animal and human [22, 23, 34, 39], which has a well-defined structure of osteons joined by interstitial lamellae, whereas the current study encompassed trabecular bone. It has been suggested that the average mineral content and crystallinity of homogenised cortical bone does not vary with age, even though individual components do exhibit age-related changes [39] because age-related changes in the primary lamellar bone which is formed in the latter stages of puberty and remains present throughout the lifespan of an individual, may be negated by the remodelling that occurs in the secondary osteons [39] in terms of measurement of overall composition. Trabecular bone does not have a component that is equivalent to primary lamellar bone, it undergoes constant remodelling and therefore has a high degree of variability in terms of chemical composition which may provide an explanation for the present inconclusive results.

### Limitations

The study represents only a small-scale assessment of the use of horse-bone sources to examine BMOA. In addition, the sample population used for these studies was heterogeneous, with very limited demographic information. Hence it was not possible to consider the effect on the measured data of horse breed, sex or working history. It was possible to consider the effect of horse age (see above), but these data require cautious interpretation as ageing a horse using dental examination is not precise [18]. Furthermore, the study did not attempt to evaluate or grade the tissue samples for osteoarthritis. However, despite these limitations, the study provides evidence that differences in bone density are associated with BMOA, suggesting that the techniques of the present study may provide useful avenues for further exploration.

### Future work

Intra-vital studies have previously demonstrated that fracture healing in bone can also be assessed with Raman microspectroscopy by measuring the lipid and phospholipid present in cell membranes that are a marker of cell death, although quantifying the presence of blood products is more difficult due to structural modifications associated with exposure to the laser [22]. As a supplement to the current study, such techniques could be used to examine whether BMOA lesions are associated with micro-damage. However, this would require the development of better methods for sample handling – in the present study, the bone marrow could not be preserved due to the method of sample preparation and storage (under saline) and blood breakdown in the time between euthanasia and Raman data collection would have resulted in spectrum modification.

Combining Raman microspectroscopy in a controlled equine population, alongside techniques such as histology or Scanning Electron Microscopy would enable information about bone remodelling to be correlated with any demonstration of micro-fracture, cracks and histological evidence of bone remodelling. Whilst there are differences between equine and human bone it is also felt that there are similarities, for example in the pathogenesis of osteochondrosis [40] that may also render these findings applicable to the human population.

## Conclusion

The majority of the BMOA lesions observed in the equine metacarpophalangeal joint occurred at a characteristic location corresponding to the region of greatest loading within the joint, in a region associated with palmar osteochondral disease [29, 30]. The data presented here demonstrated an association between BMOA and locally increased bone density, without an associated change in the chemical composition of bone. The presence of increased bone density associated with BMOA does appear to suggest that an increased amount of bone formation is occurring in these regions. As the Raman microspectroscopy data do not demonstrate any significant changes in bone chemical composition associated with BMOA, it would appear that the increased bone volume is due to a greater amount of bone being formed rather than an imbalance in relation to bone remodelling. The study provides a proof of principle for the use of Raman microspectroscopy and projection radiography in in-vitro studies of BMOA. These techniques may be a useful adjunct for further investigations into the pathophysiology of equine joint disease, which may have some relevance to similar conditions in the human population.

## Additional file

Additional file 1: Raman and x-radiographic data from individual samples. (PDF 111 kb)

## Data Availability

Data generated and analysed during this study are included in this published article [and its supplementary information files].

## Abbreviations

BMD: Bone mineral density
BMOA: Bone marrow oedema-like abnormalities
MRI: Magnetic resonance imaging
PLQ: Posterior lower quadrant
PUQ: Posterior upper quadrant
SID: Source to image receptor distance
STIR: Short tau inversion recovery
w/v: weight by volume
WSR: Within slice ratio
