# Supplementary material for: Investigation of changes in bone density and chemical composition associated with bone marrow oedema-type appearances in magnetic resonance images of the equine forelimb
Source: BMC Musculoskelet Disord. 2019 Jul 15;20:330. doi: 10.1186/s12891-019-2693-y (PMC6631911; doi:10.1186/s12891-019-2693-y)
Supplement: Supplementary file 1 — Raman and x-radiographic data from individual samples. (PDF 111 kb) [file 12891_2019_2693_MOESM1_ESM.pdf]

Within Slice Ratios

		Area under the Raman peak			Raman Band Centre	Projection radiography
Sample Code	Evidence of BMOA on MRI?	carbonate:phosphate	carbonate:amide I	phosphate:amide I	Phosphate	Mean bone density
S12 M	yes	0.6373	0.9186	1.0521	0.9998	1.192676624
S12 L	yes	1.3633	1.2353	0.9797	1.0004	1.163069819
S13 M	yes	0.8181	0.9475	1.0836	0.9998	1.20344874
S18 M	yes	0.5909	0.6620	1.0068	1.0004	1.296333417
S21 M	yes	0.9651	0.9771	0.9778	1.0002	1.321410537
S21 L	yes	0.8177	0.8578	1.0389	1.0000	1.380811561
S33 M	yes	0.7189	0.8924	1.1352	1.0001	1.238252891
S33 L	yes	0.8496	1.1108	1.2927	1.0000	1.157185587
	Mean	0.8451	0.9502	1.0709	1.0001	1.2441
	Standard Deviation	0.2415	0.1706	0.1042	0.0002	0.0809
	Standard Error	0.0854	0.0603	0.0368	0.0001	0.0286
S10 L	no i.e. control sample	1.1327	1.0296	0.9486	1.0005	0.861761243
S13 L	no i.e. control sample	1.0460	1.1193	0.9416	0.9989	1.084517344
S14 M	no i.e. control sample	0.6545	0.9433	0.9345	0.9998	1.070310723
S14 L	no i.e. control sample	0.9810	0.9070	0.9444	0.9999	1.089823882
S15 M	no i.e. control sample	1.1332	1.0447	0.9199	1.0003	1.369422687
S15 L	no i.e. control sample	0.8854	1.2099	2.3103	0.9998	0.905880372
S16 M	no i.e. control sample	0.6959	0.7392	0.9279	1.0000	1.009858635
S16 L	no i.e. control sample	0.8812	0.9224	1.0491	0.9999	1.263743901
S17 M	no i.e. control sample	0.9797	1.0693	1.0800	1.0000	1.230741331
S17 L	no i.e. control sample	0.6808	0.6244	0.9093	1.0000	1.369464694
S18 L	no i.e. control sample	0.8797	1.2234	1.2161	0.9999	0.817901325
S19 M	no i.e. control sample	0.8964	0.9633	0.9947	1.0000	1.108641199
S19 L	no i.e. control sample	0.7563	0.7359	0.9110	1.0000	1.325691387
S22 M	no i.e. control sample	0.3140	0.5710	1.0722		1.241523488
S22 L	no i.e. control sample	0.3501	0.5710	1.0357		0.594596859
S24 M	no i.e. control sample	1.5913	1.1376	0.7168	0.9999	0.869943905
S24 L	no i.e. control sample	0.8582	0.7294	0.7939	0.9999	1.128391996
	Mean	0.8657	0.9142	1.0415	0.9999	1.0790
	Standard Deviation	0.2979	0.2164	0.3458	0.0003	0.2158
	Standard Error	0.0722	0.0525	0.0839	0.0001	0.0523
	t-test	0.8565	0.6579	0.7519	0.2132	0.0111
